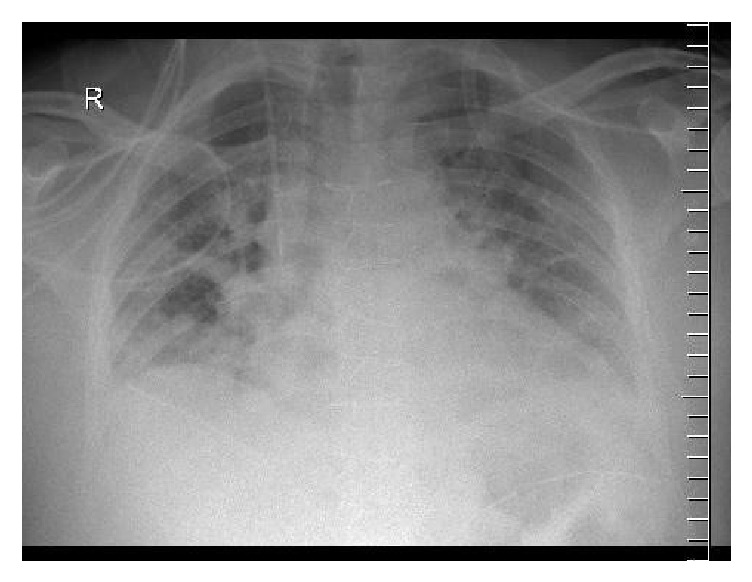# First Confirmed Case of Middle East Respiratory Syndrome Coronavirus Infection in the Kingdom of Bahrain: In a Saudi Gentleman after Cardiac Bypass Surgery

**DOI:** 10.1155/2017/1262838

**Published:** 2017-08-28

**Authors:** Nahed Seddiq, Manaf Al-Qahtani, Jaffar A. Al-Tawfiq, Nazar Bukamal

**Affiliations:** ^1^Department of Medicine, Bahrain Defence Force Hospital, Riffa, Bahrain; ^2^Royal College of Surgeons in Ireland, Bahrain Medical University, Muharraq, Bahrain; ^3^John Hopkins Aramco Healthcare, Dhahran, Saudi Arabia; ^4^Indiana University School of Medicine, Indianapolis, IN, USA; ^5^Mohammed Bin Khalifa Cardiac Centre, Riffa, Bahrain

## Abstract

Middle East Respiratory Syndrome Coronavirus (MERS-CoV) is well known to cause severe respiratory infection and was first reported in the Kingdom of Saudi Arabia in 2012. We report here the first confirmed MERS-CoV infection in the Kingdom of Bahrain in a Saudi gentleman who was admitted electively for coronary bypass surgery, postoperatively developed an acute respiratory illness, and tested positive for MERS-CoV. 40 close contacts, all healthcare workers, were traced and followed with no documented secondary cases.

## 1. Introduction

Middle East Respiratory Syndrome Coronavirus (MERS-CoV) causes severe human infections, resulting in a high mortality rate, and it has the ability to transmit between humans [1]. There remains a lack of evidence of sustained human-to-human transmission in the community [2]. Thus far, the observed human-to-human transmission has mainly occurred in the health care setting [3].

Human infection with MERS-CoV was first observed in Jeddah, Saudi Arabia, in September 2012 in patients with severe pneumonia [4]. The first hospital outbreak was then reported in a public hospital in Al Zarqa, Jordan, and involved 8 healthcare workers [5]. As of 10 March 2017, 1,979 laboratory-confirmed cases have been reported to the WHO with 684 mortalities in 27 countries [6].

Here we report the first confirmed MERS-CoV case in the Kingdom of Bahrain in a gentleman coming from the Kingdom of Saudi Arabia (KSA) who was electively admitted to Sh. Mohammed Bin Khalifa Bin Sulman Al Khalifa Cardiac Centre (MKCC) for coronary bypass surgery and developed severe respiratory illness 2 days after his surgery. The surveillance, contact tracing, and follow-up for the 40 exposed healthcare workers revealed no evidence of a secondary case.

## 2. Case Report

**Figure 1:**
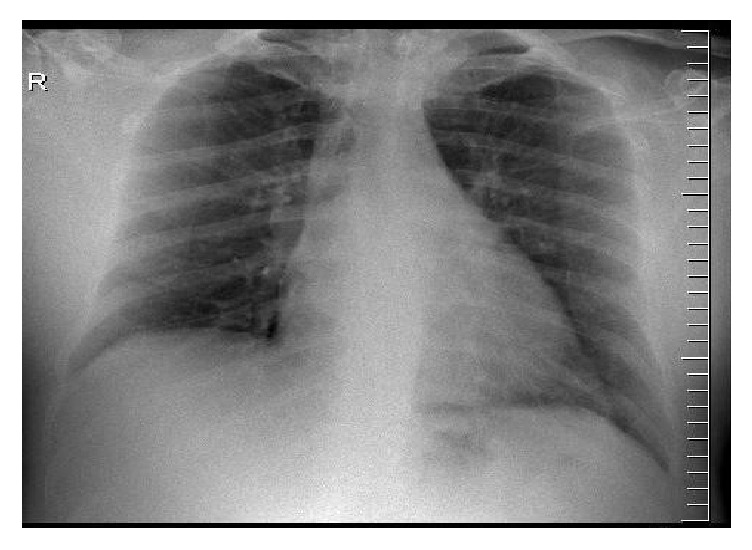


A 61-year-old male, who was originally from KSA and had a history of hypertension and diabetes without smoking, is reported here. He was diagnosed with triple vessel disease in the Aramco hospital in KSA and was advised to undergo coronary bypass surgery. He was electively admitted to MKCC on the 29th of March 2016 for surgery. At the time of admission, he was asymptomatic. He was initially screened by a nasopharyngeal swab for MERS-CoV per protocol and tested negative. The patient owned a dromedary camel barn in Saudi Arabia and had a history of close contact with camels as well as of consuming their raw milk for an unknown exposure duration. He had no other significant symptoms, risk factors, or exposures prior to admission. His initial chest X-ray on admission was unremarkable (Figure 1), and his basic laboratory tests were normal including liver function tests (white blood cells 6 × 10^9^/L, haemoglobin 13 g/L, platelets 190 × 10^9^/L, neutrophils 42%, aspartate transaminase 23 IU/L, alanine transaminase 39 IU/L, alkaline phosphatase 59 IU/L, gamma-glutamyl transpeptidase 40 IU/L, urea 4.5 mg/dL, and creatinine 103 g/dL).

On April 3, 2016, he underwent coronary artery bypass graft surgery (CABG) with no complications. After the operation, he was transferred to the cardiac intensive care unit (CICU) in a general bed with no isolation and was successfully extubated the next day. His condition was sufficiently stable to be downgraded to the high dependency unit (HDU), which is a step-down unit in the cardiac ward.

On April 5, 2016, the patient was noted to be febrile (temp 38.5°C) with mild hypoxia managed by noninvasive ventilation, and his CXR showed a few bilateral infiltrates.

On April 6, 2016, he was continuously spiking a fever up to 40°C with worsening hypoxia; in response, he was intubated and mechanically ventilated. He was observed to have worsening of his CXR (Figure 2). The next day, he was on maximum ventilatory support and required extracorporeal membrane oxygenation (ECMO). He developed acute kidney injury for which Continuous Venovenous Hemofiltration (CVVHF) was initiated. His liver function tests as well were deteriorating. During this period, all of his studies were negative for routine pathogens, including blood cultures, urine, ET aspirate culture, and respiratory panel, and he was empirically maintained on Tazocin. Meropenem was later added.

On April 9 (day 6 post-op), he had not improved. In view of his risk factor for MERS-CoV exposure, a repeated nasopharyngeal swab and deep ET aspirate were obtained. Three specimens, including a nasopharyngeal swab, deep endotracheal aspirate, and serum sample, were collected and tested using the MERS rRT-PCR assay. The results confirmed MERS-CoV infection. On 12 April, he was transferred to a hospital in Dammam, Saudi Arabia. Later that day, he passed away.

### 2.1. Infection Control Measures

On April 9, 2016, at 8 PM, the public health laboratory of the Bahrain Ministry of Health was contacted (MKCC) to confirm the positive results of MERS-CoV. At 9:30 PM, a task force team with BDF and MKCC was formed and was on site for situation assessment. The index patient was isolated and all exposed patients were identified and immediately isolated as well. Surveillance for all exposed healthcare workers was performed. Detailed environmental surveillance was also performed, and four areas with possible contamination were identified (CICU, HDU, Theatre and recovery area, and microbiology lab) and environmental swabs were taken from all four areas.

As soon as the diagnosis of MERS-CoV was confirmed, all contacts were listed. We identified 40 contacts, including physicians, nursing staff, technicians, and others. Nineteen were symptomatic with mild upper respiratory symptoms. All traced contacts underwent investigation. NP swabs for MERS-CoV and serological samples for MERS rRT-PCR assay were obtained at days 0, 14, and 28 from the date of last known exposure to the confirmed case. Since the exposed cases were mostly healthcare workers, investigations included detailed information on type of contact with the patient, protection used during the contact, and procedures done for the patient during hospitalization.

The infection control team followed all contacts for a period of 14 days for any clinical symptoms, and a hotline was available for them to contact infection control officers at any time in case of symptoms. Family members were requested to have a screen performed in KSA.

After thorough contact tracing and surveillance, no postexposure cases were identified.

## 3. Discussion

Coronaviruses are large enveloped single-stranded RNA viruses that cause a spectrum of clinical syndromes. MERS-CoV is considered the sixth coronavirus that may infect humans [4]. MERS-CoV is thought to be a zoonotic virus with transmission from an animal reservoir to humans. Several studies in the Gulf area have shown a close genetic relationship between human and camel MERS-CoV [7] and have demonstrated that human MERS-CoV infection may be directly acquired from camels [8].

The origin of the virus seems to be the Arabian Peninsula since most of the clusters and outbreaks are reported within the same area and 80% of cases are from KSA [9]; the affected population either is in close contact with camels or acquired the infection in the healthcare setting.

All MERS-CoV outbreaks outside of the Arabian Peninsula had index cases imported from that region. Recently, an unexpected outbreak occurred in Korea that was considered the largest such outbreak outside of the Middle East area. There were 187 confirmed cases, and the outbreak resulted from an imported case with a history of travel to the Middle East [10].

There have been no reports of sustained human-to-human transmission in the community. Household transmission is limited for unclear reasons, and nosocomial transmission is well known in healthcare settings in most of the reported clusters and outbreaks [9].

There are ongoing investigations to determine the mode of MERS-CoV transmission in healthcare facilities since the secondary cases vary in their degree of contact and the role of environmental contamination has been studied in a few reports. Interestingly some studies reported the presence of the virus on surfaces (patient rooms) following discharge or death, which highlights the importance of strict and proper infection control practice in healthcare facilities [11].

The latest CDC recommendation is to collect multiple specimens from different upper and lower respiratory samples as well as a serum sample to increase the likelihood of detecting the virus, and a deep respiratory sample has always been preferred [12]. In one study on 63 patients in the ICU, MERS-CoV was only detected in 20.7% of the first nasopharyngeal samples and 76.5% of the first deep tracheal aspirate samples [13].

In our patient, the first nasopharyngeal swab was negative. At that time, he was asymptomatic. A second sample was collected after he deteriorated; this sample was an endotracheal aspirate. Serology tests with the MERS rRT-PCR assay are not routinely used, but we do not know whether such tests would have shown the presence of antibodies. Recent papers have demonstrated serological evidence of the virus in healthy humans with close camel contact [4]; the patient presented here had contact with camels and consumed raw camel milk, which supports the serological findings.

Furthermore, more studies are needed to determine whether the patient was a healthy carrier of MERS-CoV who then had the virus activated by the stress of surgery.

Questions regarding the transmission of MERS-CoV are still under investigation; human-to-human transmission has been shown in most hospital outbreaks [1]. In our case, despite having up to 40 healthcare contacts, there were no documented secondary cases after thorough surveillance.

Since the discovery of MERS-CoV in 2012 in KSA, no cases were detected in Bahrain prior to our case. This observation raises a few questions. Bahrain is linked to the eastern region of the Kingdom of Saudi Arabia by a 25-km causeway that was launched in 1986. It is considered one of the busiest bridges in the region and has traffic statistics of almost 20 million passengers both ways based on 2013 data. However, despite this heavy traffic and the proximity to an area that is considered to have the highest reported cases and outbreaks, this is the first reported MERS-CoV case from that area. The reason for this observation is not clear, and no serology tests were performed on camels or humans in Bahrain to evaluate the genetic susceptibility, antibodies, or possible carrier state. We cannot confirm whether there were undetected cases.

## 4. Conclusion

A learning point from previous disease outbreaks occurring before this more recent coronavirus discovery, such as the SARS outbreak, is that outbreaks of diseases, especially viral diseases, can be catastrophic, and they have both social and economic consequences. The importance of strict, well-organized infection control policies should be addressed nationally and internationally. The rates of nosocomial transmission of MERS-CoV are very high for reasons that are under investigation. In view of this, hospitals should upgrade their infection control measures and protocols when handling suspected or confirmed cases, which can prevent outbreaks.

We still have insufficient knowledge to fully understand the transmission mode and risk factors. Given the limited treatment options, we depend on our policies and guidelines for reducing transmission.

## Figures and Tables

**Figure 2:**